# Treatment of Devitalized Teeth

**Published:** 1897-06

**Authors:** Louis Jack


### TREATMENT OF DEVITALIZED TEETH.[1]

BY LOUIS JACK, D.D.S.

The subject of preparation of the pulp-cavities of devitalized teeth and of root-canal filling has been so constantly written upon that it is difficult to present any line of treatment possessing newness. In accepting your invitation, the only thing available is to give the methods pursued by me in daily practice.

The subject is divisible into three sections: 1. Where the pulp has been freshly devitalized; 2, old devitalizations where no infectious conditions appear to exist; 3, old devitalizations where diseased conditions are apparent.

In respect of each of these classes, the preparation of all cases require such formation of the cavity or a special opening into the pulp-chamber and canals as may give directness of approach, to permit complete access, to effect cleansing of the root-canals and their subsequent occlusion. In respect of this, as applied to the upper molars and bicuspids, the best access is found by cutting through from the occlusal surface. But when the aspect of the cavity is mesio-proximal and the cavity large, choice must rest, of opening sufficiently from that direction, as otherwise the tooth will be unnecessarily weakened. Should the cavity be on the distal aspect, thorough preparation requires opening from the occlusal surface. The same principle of conserving the strength of the tooth applies to the incisors and cuspids. When they are strong, the approach is best from the lingual aspect, and where weak from the carious cavity.

Concerning the inferior molars and bicuspids, when the cavities are on the mesial proximate surfaces, the choice rests to open here with sufficient freedom. When the cavity of decay is on the distal surface of these teeth, the best approach is from the buccal aspect

---

[1] Read before the New York Institute of Stomatology, March, 1897, at a point near the cervix. In respect of this choice it is not necessary to make a large opening, since, by extending it laterally in each direction and also downward, the distal canal, and also the mesial one, can be easily opened, and may be filled through a comparatively small opening in the molar, and with the bicuspid, by drilling inward and downward, much less loss of structure is caused than any attempt to approach from the distal surface. Nothing so weakens a lower bicuspid as free cutting on its distal aspect. Having covered, in this general way, free approach to the root-canals, I will now consider the question of treatment applicable to each of the class of cases above cited.

Where the pulp has been recently devitalized, the chief considerations are, after the removal of all remains of the dead pulp, to preserve the most absolute cleanliness, to avoid the infection of the canal by the germs always present in the mouth, and the avoidance of infection by instruments which are tainted. It hence follows that the fluids of the mouth should be excluded, and that either new or thoroughly disinfected instruments only should enter the canals. I am careful, in the removal of the pulp, to use new Swiss broaches, the temper of which I immediately draw to a gray color and use, or if not absolutely fresh, I pass them through the flame; but as this is liable to burn the finer ones, it is generally preferable to take a new broach. There being no infection, there is no reason against the immediate filling of the canal.

Concerning the question of trouble ensuing to the peridental membrane from the putrefaction of the contents of the canaliculi, I have taken little account of, except in the teeth of young children, where I sometimes dress the canal with a twenty-grains to the ounce solution of zinc chloride. In all cases, however, I finally dress the canal with a saturated solution of aristol with gaultheria.

In respect to Class 2, there is no question of the necessity of approaching them with great caution, since, whether the tooth is without caries or has a cavity of decay, the greatest danger is of infecting the tissues of the apical region. This I believe is very frequently caused by injecting some portion of the contents of the canal through the foramen. This may be effected either by making compression by the use of large instruments in opening the cavity, or by inserting instruments within the chamber or canal previous to disinfection of their contents. My practice is to make a small opening into the chamber by drilling, or otherwise, and inserting a small crystal of potassium permanganate, allowing it to dissolve in the fluids there, or adding a modicum of water if the canal is dry.

In respect to the use of permanganate, it is important, in the front teeth, to use a weak solution, or to employ formalin, for the reason that there is danger of some discoloration of the dentine by the oxide of manganese. This is more particularly liable to occur with young children. When a portion of the detritus of the pulp is removed, I carefully convey on a broach a weak solution of permanganate.

I then close the canals with a thread filled with the solution of aristol before named, seal the outer cavity, and await indications of the possible previous infection of the apical tissues. If in forty-eight hours these do not appear, I proceed to fill the canals.

In respect to the third class, the subject is so complex that it is difficult in such an effort as this to go over the ground in detail, for the reason that the conditions are so various, in consequence of the varying impressibility of the tissues of different persons to infectious influences, and also probably because in different mouths the pathogenic character of the organisms vary in their virulence.

When there is a fistula and an open foramen, or where we can safely and surely make a small enlargement of the foramen, the case is reduced to simplicity, since a single irrigation of the fistulous tract with zinc chloride, five to ten grains to the ounce, or of ninety-five-per-cent. carbolic acid, will usually be sufficient to produce a cessation of the purulent discharge.

When, however, there is no fistula, the difficulty of disinfection is increased. My plan here is to expect a correction of the conditions by the slow admission of a disinfectant which in its nature is not irritating. For this purpose I loosely fill the canal and pulp-chamber with lint saturated with the solution of aristol, and fill the outer cavity around a root-filling instrument, of such size as will permit the effusions forming at the apical region to escape through the small opening left on the withdrawal of this broach.

The case is then repeatedly dressed in this manner, each time reducing the size of the broach-opening until at last its use may be avoided. Then I temporarily fill the canal tightly with a thread dipped in aristol as a tentative measure.

There is one class of cases of this kind where there is no effusion, but which will not tolerate closure. At each attempt irritation sets in, with threat of inflammatory conditions. I have found with these the greatest benefit to follow the use of a two-and-a-half-per-cent. aqueous solution of formalin, with which the canals are dressed. It is important to avoid forcing formalin through the foramen, since it excites a degree of irritation scarcely explainable. Neuralgic soreness arises, not attended by inflammatory conditions. This condition does not easily abate. Therefore I throw out this caution. In passing, I may state that in the treatment of apical disorders there has too often been exhibited the disposition to use active medication when patient administration of milder disinfectants would be more efficient. After the active disturbances have passed, I continue the disinfection with aristol in one of the essential oils.

*Canal Filling.*—Concerning the filling of the canals, I generally air-dry them, and coat the surfaces with chloro-percha and insert hard cones of gutta-percha. In some cases I employ oxychloride of zinc. In large roots I feel it important to mallet into the apex a cone of gold-foil to perfectly occlude the end of the canal. With reference to the buccal roots of upper molars and the mesial roots of the inferior molar when it is not feasible to enlarge them, I rely upon the instillation of chloro-percha or oxychloride of zinc, but have to confess, in many cases, inability to either remove all the remains of the pulp or to secure perfect occlusion.

In conclusion, I would state that where there is reason to believe the canals have been filled when after-disturbance occurs, which does not at once yield to refrigeration and the application of iodine, I perform alveolotomy. The technique of this operation is to refrigerate the gum over the end of the root, puncture the gum, and drill directly into the apical region, making a small aperture. Before drilling, the gum-tissue at the puncture is pressed upward in order that the retraction of the gum will cover the drilled orifice.

The result of this operation usually immediately aborts inflammatory conditions by depletion, and also brings on irrigation of the apical tissues by the flow of blood. The result of alveolotomy where indicated is followed by salutary results.

